# Oral health assessment in a prospective birth cohort study

**DOI:** 10.1038/s41405-025-00395-9

**Published:** 2026-01-16

**Authors:** A. M. Kaan, D. D. Duijster, J. K. Ujcic-Voortman, L. V. Haring, C. M. C. Volgenant, E. Zaura

**Affiliations:** 1 Department of Preventive Dentistry, Academic Centre for Dentistry Amsterdam (ACTA), Vrije Universiteit Amsterdam and University of Amsterdam, Amsterdam, The Netherlands; 2 Department of Healthy Living, GGD Amsterdam, Amsterdam, The Netherlands; 3 Department of Oral Public Health, Academic Centre for Dentistry Amsterdam (ACTA), Vrije Universiteit Amsterdam and University of Amsterdam, Amsterdam, The Netherlands; 4 Sarphati Amsterdam: Research for Healthy Living, Amsterdam, The Netherlands; 5 Department of Cariology, Academic Centre for Dentistry Amsterdam (ACTA), Vrije Universiteit Amsterdam and University of Amsterdam, Amsterdam, The Netherlands

**Keywords:** Oral diseases, Dental public health

## Abstract

**Background:**

Robust oral health data collection in birth cohort studies is needed to understand the oral microbiome in relation to oral and general health.

**Objective:**

The aim of this paper is to describe the collection of oral health data in toddlers participating in a birth cohort focussing on microbiome development. Hereby, we aim to support the interpretation of variance in microbiome data.

**Methods:**

The Amsterdam Infant Microbiome Study (AIMS, *n* = ~500) is a longitudinal prospective birth cohort assessing microbiota, general health status, demographics, (oral) health behaviour and dietary behaviour in children from birth up to three years. The Oral Health Study (OHS) is a sub-study of AIMS, assessing the oral health of children and their mothers. From the mothers, data on periodontal health (clinical attachment loss, gingival bleeding), oral hygiene (dental plaque, calculus) and dental caries (DMFS) is collected. In children, data on caries prevalence (ICDAS) and infection (pufa), oral hygiene (dental plaque, calculus), Obstructive Sleep Apnoea (OSA), oromuscular function, and bitter taste sensitivity are collected.

**Results:**

Enrolment in OHS started in October 2022 and is planned to continue up to December 2028. In October 2024, 64 mother-child pairs were enroled in the study.

**Conclusions:**

Data collection is expected to be completed by January 2028. Results will be shared at international conferences and via peer-reviewed publications.

## Introduction

Oral health is an integral part of general health and oral diseases are among the most prevalent public health issues worldwide [1]. Dental caries and periodontitis are among the most prevalent non-communicable diseases worldwide, along with diabetes, metabolic diseases and cardiovascular diseases [2]. Links between oral diseases and the severity of specific non-communicable diseases have been established [3]. The effect of oral hygiene practices, including toothbrushing and interdental cleaning, on reducing the risk of developing type 2 diabetes or hypertension was established in a recent systematic review, and periodontal therapy has been shown to reduce cardiovascular risk biomarkers [4, 5]. During pregnancy, periodontal disease can worsen insulin resistance and promote the development of gestational diabetes mellitus [6]. As the consequences of poor oral health during childhood may track into adulthood, early-life oral health problems pose great concern for health throughout the lifespan. Dental caries is estimated to affect 46% of the children under the age of six years, globally and is known to negatively affect school attendance, educational performance, and growth and development [7–9].

Dental caries is a complex and multifactorial non-communicable disease, influenced by various biological, behavioural, psychosocial and environmental factors, each of which has been extensively studied [10]. One of the less explored aspects is the relation of gustation (taste) with dental caries. It has been reported that bitter taste receptors are associated with severe early childhood caries [11]. The ability of an individual to detect bitterness by tasting is called bitter taste sensitivity. Sensitivity for bitter tastants is associated with liking of sugary foods [12]. Specifically, the TAS2R38 gene causes insensitivity to bitter compounds such as phenylthiouracil (PTC) and 6-n-propylthiouracil (PROP) [12], which substances have been used to identify bitter taste insensitivity in scientific studies. Apart from their effect on taste preference, oral taste receptors that detect bitter tastants may influence caries through mediation of oral host-microbiome interaction [13]. The TAS2R bitter taste receptor family is involved in innate immune responses to bacteria, including the caries-associated bacterium *Streptococcus mutans*. Specifically, TAS2R14 responds to a quorum-sensing molecule secreted by *S. mutans* and consequently induces a rapid innate immune response [14]. Also, the oral microbiota has been suggested to influence flavour perception [15]. The influence of sensitivity for bitter taste in dietary preferences at a young age, however, is not yet fully understood, as food choices are not only based on sensitivity for specific taste modules, but also on other factors such as exposure to specific foods by parents or caretakers, olfactory stimuli, colour, and texture of the food, and behavioural aspects of eating [16–18].

Another understudied aspect is the role of orofacial myofunctional development on dental caries. Habits such as mouth breathing, tongue thrusting, thumb sucking and reverse swallowing can influence jaw development and have been associated with orofacial growth abnormalities and several oral health problems, such as dental caries and gingivitis [19, 20]. Most growth of the maxillofacial structures takes place during the first 4 years of life and during puberty [21]. However, to our knowledge, no longitudinal studies on toddlers assess the relationship between myofunctional oral habits and oral health outcomes.

Oral health is usually dealt with in isolation from the rest of the body, while there is a growing consensus that oral health has an impact on overall health and should be addressed as an integral part of overall health [22]. In children, dental caries and overweight are shown to correlate, while early weight gain is shown to forecast not only overweight but also to accelerate the eruption of deciduous teeth and risk for dental caries [23, 24]. Additionally, oral microbiota composition has been associated with rapid infant weight gain [25].

To understand the biological processes involved in the aetiology and course of non-communicable diseases from childhood onwards, several longitudinal birth cohort studies have been initiated. These types of studies are suitable for clarifying links between early life development and non-communicable diseases, or for understanding the relation between oral microbiome and (oral) health. For example, a multidisciplinary prospective cohort study – the Amsterdam Infant Microbiome study (AIMS) (www.aimsonderzoek.nl) – has been initiated by the Public Health Services (GGD) of Amsterdam, the Netherlands, aiming at understanding the interplay between the microbiota and health in the first three years of life.

In most of the cohort studies addressing gut and oral microbiome, collection of oral health data is not included, performed in a way that limits detection of early-stage caries [26, 27] or includes self-reporting of oral factors that could influence the microbiome [28, 29]. In a subcohort of AIMS, called Oral Health Study (OHS), oral health-related data is being collected. The purpose of the OHS is to support the interpretation of the microbiome data from a biological point of view.

The aim of this paper is to describe the collection of oral health data in toddlers participating in a birth cohort focussing on microbiome development. With this, we aim to support the interpretation of variance in microbiome data.

## Methods

Here, we describe methods on how to collect oral health data in toddlers and their mothers participating in a longitudinal birth cohort. The collected oral health data is to be combined with parameters collected in associated studies. Analysis of the combined data is not part of this paper and will be produced separately.

### Embedding of the Oral Health Study in other studies

**Fig. 1:**
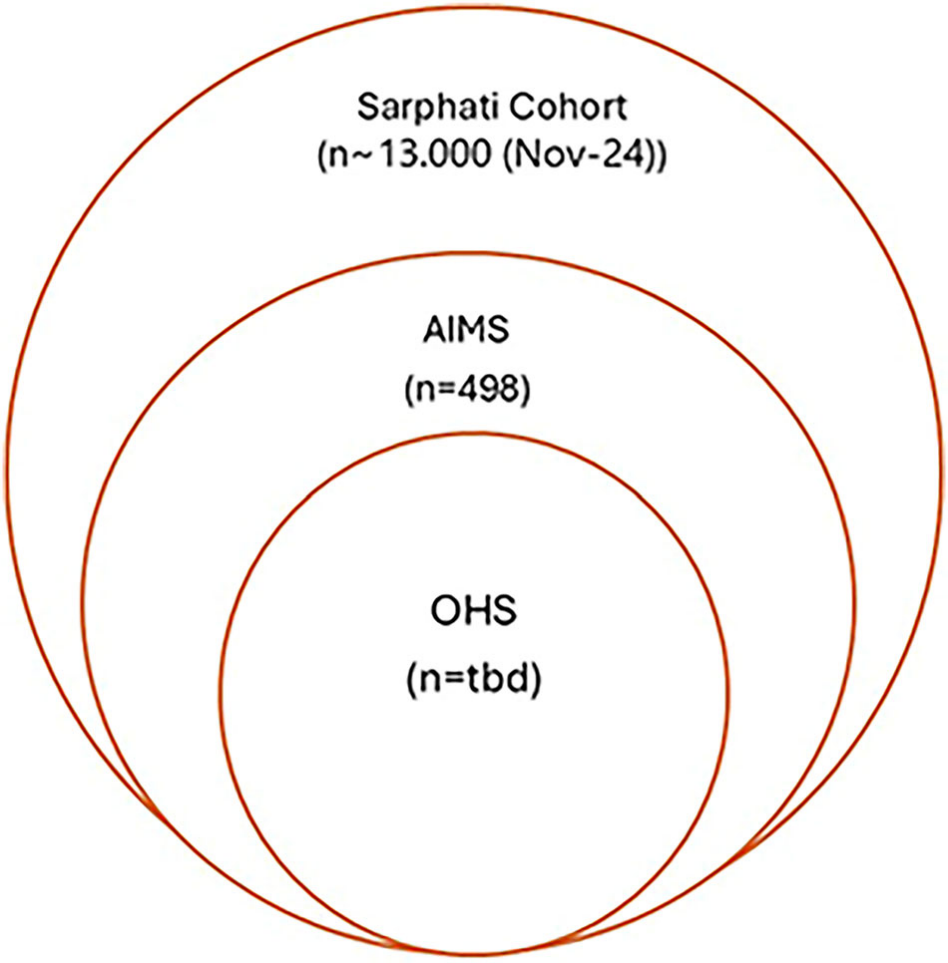
Embedding of the OHS in large-scale longitudinal cohort studies.

OHS is a subcohort of the Amsterdam Infant Microbiome Study (AIMS) (https://aimsonderzoek.nl), which in turn is a sub cohort of the Sarphati Cohort (https://sarphaticohort.nl) (Fig. 1). The Sarphati Cohort was initiated in 2018 as a collaboration between the Public Health Service (GGD) of Amsterdam and several knowledge institutions, as an initiative to contribute to healthy growth and development and to reduce health disparities among Amsterdam residents. The Sarphati Cohort is an open (dynamic) population-based prospective cohort study. It aims to study the development and health of children growing up in Amsterdam in relation to lifestyle and living environment. Data is collected during their first 18 years of life by questionnaires and during regular consultation visits at the Youth Healthcare Centre (JGZ). AIMS is a longitudinal birth cohort study of 498 families, in which data is collected during pregnancy and the three years following the birth of their child. Participants were recruited between May 2019 and November 2024. Inclusion criteria for AIMS are mothers who are: 1) aged 18 years or older; 2) maximum 34 weeks pregnant; 3) living in Amsterdam and not planning to move out of Amsterdam within three years. Data collection includes biosamples (tongue swab, supragingival plaque, stool, vaginal swab, and breastmilk) for microbial analyses and questionnaires addressing health status, sociodemographics, (oral) health behaviour and dietary behaviour. The study protocol is currently being drafted for publication in a scientific journal. The Sarphati Cohort and AIMS are executed by the Public Health Service of Amsterdam.

### Ethical considerations and recruitment

The research protocol of the OHS has been approved by the Medical Ethics Committee of Amsterdam Medical Centre (NL64399.018.17, 15-06-2022) as an amendment to the AIMS study protocol. STROBE guidelines were consulted and used where appropriate due to the observational nature of the OHS. The OHS was registered in the Open Science Framework repository (OSF) (https://osf.io/fm3ga). Participation is voluntary and participants can withdraw at any moment without any consequences. Also, participation of either the mother or the child is possible. All families in AIMS are eligible for participation in OHS. Mothers or legal guardians participating in AIMS are invited per email to participate in the OHS when their child is approximately 1.5 years old, and again when their child is about 3 years old. Through an online survey, parents can give permission to send them additional information about the OHS and/or give permission to the researcher-dentist to call them. When they give permission, written information about the study is sent to them by postal mail, and they are contacted by phone to answer any questions. If they agree to participate, written informed consent is obtained and an appointment is made for the clinical oral examination for both the mother and the child (participation of either the mother or the child is also possible). This clinical oral examination takes place in a dental practice located in one of the six areas in the city of Amsterdam. All participants are offered oral hygiene materials, including a toothbrush and toothpaste and personalized oral hygiene advice as a token of appreciation for their participation. Data collection for the OHS started in October 2022 and is currently ongoing.

### Clinical oral examination of the child

During the clinical oral examination of the child, parameters concerning oral hygiene, oral health, oromuscular function, and bitter taste sensitivity are collected. All intra-oral examinations in children are performed with the accompanying person sitting in a dental chair and the child sitting on the lap of the accompanying person. If the child is unwilling to sit in the dental chair, the child can lie down on a changing mat, or on the legs of the accompanying person and the examiner in a knee-to-knee position. First, the amount of dental plaque is assessed using the Quigley and Hein index and the amount of calculus using the Simplified Calculus Index (CI-S) [30–32]. The covered surface of all six tooth surfaces is scored, as well as the amount of supragingival calculus on a 4-point scale (0 = no calculus; 3 = supragingival calculus covering more than two-thirds of the exposed tooth surface, or a continuous heavy band of sub-gingival calculus, or both). Next, the presence of dental caries is scored according to the WHO Basic Methods for Oral Health Surveys and the International Caries Detection and Assessment System (ICDAS) [33, 34]. In brief, the teeth are cleaned with a toothbrush by the examiner, and dried with a gauze, after which ICDAS scores (2-6) are recorded by examining the teeth using a ball-ended probe and an intra-oral mirror. From this data, the number of teeth and tooth surfaces with early stages of decay (ICDAS score 2), established decay (ICDAS score 3 or higher), missing surfaces (m) and fillings (f) as dmft/s index will be computed. During the second visit, when the child is around 3 years old, in addition to the above, measurements regarding caries-associated oral infection, oromuscular function, tooth position and risk for Obstructive Sleep Apnoea (OSA) are collected. Following ICDAS-scoring, Pufa-index is used to measure dental infection as a consequence of severe dental caries (which includes assessment of pulpal involvement, ulceration caused by dislocated tooth fragments, fistula and abscess) [35]. Oromuscular function is assessed by asking the accompanying parent about parafunctional oral behaviour (bruxism, thumb or finger sucking, pacifier use, mouth breathing) and inspection by the examiner (mouth breathing behaviour, swallowing pattern, presence of a high arched palate and malocclusion such as cross bite, crowding, open bite, and overbite). Next, according to the OSA-5 screening questionnaire (Appendix A), the parent is asked to answer five questions to screen children at risk for OSA (Obstructive Sleep Apnoea) [36].

### Bitter taste sensitivity

Several methods to detect bitter taste sensitivity have been described and used in scientific literature (Appendix B). As not each method is feasible due to safety concerns of the tastants in children (PTC, PROP [37]), or potential objection of participants to applying genetic analysis, in OHS, a previously described method [15, 38, 39] is used, combining ingestion of a tastant and evaluating sensitivity based on the ingested amount and facial expression.

The bitter taste stimulus is a 0.18 M concentration of urea in bottled water, and volumes presented are limited to 15 mL. A fixed sequence of four cups (water – tastant – water – tastant) is presented to the child. To determine the ingestion, each cup is weighed before and after consumption. For each taste, analysis was restricted to infants who consumed at least 1.0 g from two cups over a sequence. The child’s liking of the stimulus is evaluated by an examiner, using a five-point scale ranging from ‘strong rejection’ (score 1) to ‘strong acceptance’ (score 5), based on the infants’ gusto-facial and naso-facial reflexes [40]. Studies with infants determined that facial reactions to tastes were innate and remained more or less unchanged into adulthood [41–43]. For example, if the child frowns, pushes the cup away and stops drinking, the examiner marks ‘1’ (strong rejection), whereas if the child smiles, displays a relaxed face, and drinks the liquid in the cup, the examiner marks ‘5’ (strong acceptance).

### Clinical oral examination of the mother

If the mother agreed to participate in the study, oral health status of the mother is clinically examined along with her child. Intra-oral examination of mothers is performed with the participant lying down in a dental chair. Oral health status is assessed according to the WHO Basic Methods for Oral Health Surveys and includes the dentition status and periodontal health. For the dentition status, each surface of each tooth (excluding third molars) is scored as 0: sound, 1: caries, 2: filled with caries, 3: filled, no caries, 4: missing, due to caries, 5: missing, for any other reason, 6: fixed replacement for a missing tooth, or 7: restauration for other reasons (facing, veneer, trauma) [33]. From this data, the number of teeth and tooth surfaces with established decay (D), missing surfaces (M) and fillings (F) (DMFT/DMFS index) will be computed. Caries (tooth decay) is defined as ICDAS-score 3 or higher. Periodontal health and oral hygiene measurements include measurement of gingival bleeding on probing, amount of calculus and dental plaque, periodontal pocket depth and clinical attachment loss are recorded for ten index teeth (17, 16, 11, 26, 27, 31, 36, 37, 46 and 47) as follows [44]. First, the teeth are dried, after which the amount of dental plaque is assessed using the Quigley and Hein index, and the presence/absence of supragingival calculus is assessed [30, 31]. Next, the pocket depth and gingival attachment level in mm from the cementoenamel junction is measured using a Williams periodontal probe. Clinical attachment loss (CAL) is computed based on these measurements. Bleeding on probing (BOP) is scored according to the Community Periodontal Index (CPI modified; presence/absence of the condition) [33].

### ICDAS Examiner training

The clinical examiner (A.M.K.) was calibrated for clinical caries detection. The inter-examiner reliability was determined against a senior examiner (C.M.V.) in two steps. First, fifteen coloured photographs were assessed, and in a second step, the assessment of three children aged between 1.5 and 3 years old was performed. The intra-examiner reliability was determined on 210 surfaces at least one week apart. The inter-examiner reliability will be recalibrated once a year. Additional examiners are recruited during the study and will be calibrated accordingly.

### Calibration

The intra-examiner reliability for ICDAS was 0.97 (February 2023). The inter-examiner reliability of the first calibration session (October 2023) was 0.93 for the assessment of coloured photographs and 1.0 for the clinical assessment. The annual calibration is currently ongoing, with assessment of coloured photographs planned, and clinical assessment finished (κ = 0.87).

### Study endpoints

The primary endpoint of OHS will be oral health, determined by the prevalence and severity of dental caries in children, and in mothers by the prevalence of dental caries and periodontal health (e.g., clinical attachment loss, bleeding on probing). Secondary endpoints will be parafunctional oral behaviour, OSA risk, and bitter taste sensitivity. These data will be combined with data collected in AIMS during pregnancy and the first three years after birth, regarding oral and gut microbiota, (oral) health behaviour, dietary behaviour, sociodemographics, parental (oral) health, and child growth trajectories.

### Statistics

This paper represents considerations for the collection of oral health data as part of a larger observational study. Analysis of the collected oral health data in OHS is to be combined with data collected in AIMS. The analytical methods are still ongoing and will therefore be described separately. Documentation of the oral health data as described above will be as follows. First, BOP-percentages will be calculated by dividing the number of bleeding sites by the total number of sites and multiplying this by 100. Plaque score (%) will be calculated accordingly. Parafunctional oral behaviour, bitter taste sensitivity (taster vs. non-taster), and OSA-risk (high risk vs. low risk) will be documented at the nominal level.

## Results

The first mother-child pairs were included in October 2022. Since the inclusion of pregnant women for the AIMS cohort (*n* = 498) was completed in November 2024, recruitment for OHS is expected to continue up to December 2028. During the first 1.5 years of inclusion for the OHS (October 2022–February 2024), the inclusion rate was 27%. Attempts aiming to increase the inclusion rate were made: the OHS was brought to attention by the AIMS team during in-person visits with AIMS participants as part of the AIMS study, and information about the study was added to the newsletter distributed among AIMS participants. During the following period (March 2024–September 2024) inclusion rate increased to 37%, with 64 mother-child pairs included in total. Next steps to further improve the inclusion rate will be to inform AIMS participants about the progress of OHS in a newsletter that is spread among AIMS participants on a regular basis. Completion of data collection is expected in January 2028.

### Calibration

The intra-examiner reliability for ICDAS was 0.97 (February 2023). The inter-examiner reliability of the first calibration session (October 2023) was 0.93 for the assessment of coloured photographs and 1.0 for the clinical assessment. The annual calibration is currently ongoing, with assessment of coloured photographs planned, and clinical assessment finished (κ = 0.87).

## Discussion

In this paper, we described considerations for the collection of oral health data as part of a larger observational study in families with a newborn baby, examining oral health of the child and, if agreed to participate, also its mother during the first three years of the child’s life. The described oral health assessment (OHS) is part of a longitudinal cohort study (AIMS). The oral health data collected in OHS is to be combined with data on microbiota, growth trajectories and questionnaire data on (health) behaviours, dietary behaviour, (parental) health status and sociodemographics collected in the AIMS cohort. The analytical methods are still ongoing and will therefore be described separately. To our knowledge, this is the first longitudinal prospective birth cohort study on the relationship between microbial, behavioural, demographic and parental health factors and the development of oral diseases and obesity.

The longitudinal study of the acquisition and establishment of microbiota requires extensive data collection. Loss to follow-up could reduce the number of participants finishing the study due to its longitudinal nature. In an attempt to limit loss to follow-up, only participants who were not planning to move out of Amsterdam at the time of recruitment were included. Another potential limitation could be that selection bias occurs, as complying with such study protocols requires the time and effort of the participants. Although many efforts were taken to make participation in this study as easy as possible (e.g., sample collection at home), still, complying to the AIMS protocol, including the OHS, requires considerable long-term commitment from the participants. To name a few challenges, participating in AIMS requires good planning skills, as biosamples need to be collected and stored in a specific way, during a specific period of time. Also, during the first year of an infant’s life, the data collection frequency is high, with a total of 7 measurement points. In AIMS, this may lead to selection bias. Specifically for the OHS, selection bias could occur if parents decline participation due to dental anxiety, which is reported to occur in between 24 and 40% of the Dutch population [45, 46]. Higher levels of dental anxiety have been associated with poorer oral hygiene practices [45]. As the equipment used during the clinical collection of oral health data influences the quality of the data, we found data collection in a setting other than a dental practice not favourable, although this could have increased participation of this specific patient group. To encourage participation and to limit the effect of parental dental anxiety, the study protocol allows participation of the child only.

Several methods for caries presence and severity evaluation have been used previously, ranging from caries detection at cavitation level using the WHO criteria, and using ICDAS criteria, by which also non-cavitated lesions are detected [47, 48]. The use of different indices complicates the comparison of the results of different studies; however, in previous research, it was reported that examination using ICDAS-II was comparable to the WHO criteria when the cut-off point was set at ICDAS-3 (cavitated lesions) [49]. As inclusion of non-cavitated lesions obviously increases the number of carious lesions found, the use of the ICDAS scoring system may detect associations more sensitively, which enables follow-up of de- and remineralization of the detected non-cavitated lesions. Therefore, the ICDAS score was considered the most appropriate method to identify dental caries in children in this population.

Although the golden standard for caries assessment is by clinical examination by a dentist, as an alternative, the collection of intra-oral photographs followed by caries scoring by a trained dentist has been proposed as a practical alternative. This approach was used in a longitudinal birth cohort study, and the method showed good inter-observer reliability (0.72 – 0.80) and good sensitivity (85.5%) and specificity (83.6%) [26, 50]. Potential disadvantages of this method may be that the caries detection is limited to the visible surfaces compared to clinical visual examination, and that it requires high-quality photographs and thus, cooperation of the study participant. The authors describe that out of 8,305 six-year-olds, 1003 participants were excluded due to insufficient quality of photographs [26]. Also, in 152 cases, no dental photographs could be taken, and in 208 cases, only frontal photographs were collected, precluding caries detection in the premolar and molar region [26]. Applying this method to 18- and 36-month-olds will not be feasible due to their young age and argued that clinical examination of dental caries remains the method of choice for toddlers. A potential limitation of this approach is that caries is assessed only up to three years of age. As the initiation and progression of dental caries is a process that takes several months before caries becomes clinically visible, the caries incidence at the proposed length is expected to be limited. However, low caries rates in our samples at endline do not obstruct our aim to understand oral microbiome acquisition and maintenance in relation to oral health. However, to study research questions aiming to compare caries-active and caries-free children, the current approach may be adjusted by adding caries assessment at a later age.

Our study is one of the few birth cohort studies in which, upon consent, the oral health of both toddler and mother is clinically assessed. Especially in the first years of life, the family plays a fundamental role in the initiation and maintenance of children’s oral health-related behaviour. Children aged between 2 and 3 years old whose mothers had a higher caries experience were of greater risk of having early childhood caries compared to mothers had low caries experience [47]. Combined clinical oral health data of mother-child pairs allows us to longitudinally investigate intergenerational oral health behaviours and dental caries during this critical period. A limitation of the described approach is that no clinical oral health data were collected from co-parents. Therefore, the role of co-parents in children’s oral health and oral health behaviours remains underexplored. The inclusion of co-parents could be considered in future work.

Besides child and maternal oral health, the OHS evaluates myofunctional habits that have not been evaluated in previous birth cohort studies as potential risk factors for the development of caries. We expect that the data obtained from the myofunctional assessment will provide valuable insights on the relationships between habits such as mouth breathing and thumb sucking, and oral health. In a study on 7–14-year-old children, it was found that oral, nasal and pharyngeal microbiota from mouth breathing children was different from nose-breathing children [51]. Possibly, an open mouth posture can lead to oral dryness as saliva is vaporizing easily from the oral cavity. This impedes the mechanical cleansing of saliva, resulting in higher accumulation of food debris and dental plaque, promoting aciduric and acidogenic oral microbiota and thereby increasing risk for caries development. In adults, it has been demonstrated that intraoral pH decreases and stays lower for a longer period when mouth breathing during sleep compared to nose breathing during sleep, which could be a risk factor for caries [52].

Secondly, other than previous birth cohort studies, the OHS evaluates the bitter taste sensitivity in toddlers in relation to oral health and overweight. Low sensitivity to bitter tastants is associated with a higher intake of bitter-tasting foods, including healthy foods such as vegetables, while genetic variants in genes related to bitter taste sensation have been associated with severe early childhood caries and composition of supragingival plaque [53–55]. While genetic testing can determine sensitivity to bitter taste, this was not included in AIMS due to potential concerns among participants. A limitation of the clinical bitter taste test that was used in OHS, is that it requires participants to undergo an additional procedure compared to genetic testing. The taste sensitivity method described in this manuscript is one of several existing methods for bitter taste sensitivity assessment [25]. As evaluation in toddlers is based on nonverbal factors, we selected the ingestion method, as it involves study methods (sipping) that are recognizable for the child. The proposed method in this manuscript involving evaluation of facial expression, was validated in previous research and is being used in several studies. Still, the quality of the collected data may be affected by the experience of the examiner. To overcome this issue, facial expression was calibrated between two examiners. Methods to increase the reproducibility of this method could be the use of video recordings to enable examiners to watch facial expressions more than once. Another recommendation could be to standardize the scoring of facial expressions more, for example, by aligning to the Facial Action Coding System [56, 57].

Taken together, the outcomes of the OHS subcohort will allow linkage of oral health parameters with oral and gut microbiota, (oral) health behaviour, dietary behaviour, sociodemographics, parental (oral) health, and child growth trajectories obtained in the AIMS cohort and will thereby provide valuable insights on the interplay among these factors during the first three years of life.

### Ethics statement

The research protocol of the OHS has been approved by the Medical Ethics Committee of Amsterdam Medical Centre (NL64399.018.17, 15-06-2022) as an amendment to the AIMS study protocol. STROBE guidelines were consulted and used where appropriate due to the observational nature of the OHS. The study is conducted in accordance with the Declaration of Helsinki. The OHS was registered in the Open Science Framework repository (OSF) (https://osf.io/fm3ga).

## Supplementary information

Appendices

## Data Availability

Correspondence and requests for materials should be addressed to Egija Zaura.
