# Supplementary material for: Oral health assessment in a prospective birth cohort study
Source: BDJ Open. 2026 Jan 16;12:10. doi: 10.1038/s41405-025-00395-9 (PMC12811250; doi:10.1038/s41405-025-00395-9)
Supplement: Supplementary file 1 — Appendices [file 41405_2025_395_MOESM1_ESM.docx]

**Appendix A. The OSA-5 screening questionnaire for screening children at risk of OSA.**

|  | During the past 4 weeks, how often has your child had … | None of the time | Some of the time | Most of the time | All of the time |
| --- | --- | --- | --- | --- | --- |
| 1 | Loud snoring? | 0 | 1 | 2 | 3 |
| 2 | Breath holding spells or pauses in breathing at night? | 0 | 1 | 2 | 3 |
| 3 | Choking or made gasping sounds while asleep? | 0 | 1 | 2 | 3 |
| 4 | Mouth breathing because of a blocked nose? | 0 | 1 | 2 | 3 |
| 5 | Breathing problems during sleep that made you worried that they were not getting enough air? | 0 | 1 | 2 | 3 |

**Appendix B. Overview of methods for determining bitter taste sensitivity described and used in scientific literature**

| **Method** | **Description** |
| --- | --- |
| Three-alternative forced-choice (3-AFC test)^53^ | Solutions of the taste stimulus are prepared in ascending concentrations. Each concentration is presented in a triad: one sample with the taste stimulus and two identical water samples. Participants rinse with mineral water between triads. The detection threshold is determined at the lowest concentration at which the stimulus is perceived. |
| Sequential solutions^39^ | The bitter taste stimulus is a 0.18M concentration of urea in bottled water, and volumes presented are limited to 15 mL. A fixed sequence of four cups (water – tastant – water – tastant) is presented to the child. To determine the ingestion, each cup is weighted before and after consumption. |
| Taste Strip Method^54^ | Taste strips are impregnated with ascending concentrations of the taste stimulus, plus one neutral strip. Each strip is applied to the tongue for several seconds, with water rinsing between applications. |
| Genome-Wide Association Studies (GWAS)^11^ | As bitter taste sensitivity is associated with specific taste genes (TAS2R family genes), the sensitivity for bitter tastants can be assessed by gene identification. |
